# Disulfide Crosslinked Hydrogels Made From the Hydra Stinging Cell Protein, Minicollagen-1

**DOI:** 10.3389/fchem.2019.00950

**Published:** 2020-01-23

**Authors:** Sanaz Farajollahi, Patrick B. Dennis, Marquise G. Crosby, Joseph M. Slocik, Anthony T. Pelton, Cheri M. Hampton, Lawrence F. Drummy, Steven J. Yang, Meredith N. Silberstein, Maneesh K. Gupta, Rajesh R. Naik

**Affiliations:** ^1^Materials and Manufacturing Directorate, Air Force Research Laboratory, Wright-Patterson Air Force Base, Dayton, OH, United States; ^2^UES Inc., Dayton, OH, United States; ^3^Sibley School of Mechanical and Aerospace Engineering, Cornell University, Ithaca, NY, United States; ^4^711th Human Performance Wing, Air Force Research Laboratory, Wright-Patterson Air Force Base, Dayton, OH, United States

**Keywords:** minicollagen-1, hydrogel, cysteine rich domain, disulfide crosslinking, photo-crosslinking, polyproline

## Abstract

Minicollagens from cnidarian nematocysts are attractive potential building blocks for the creation of strong, lightweight and tough polymeric materials with the potential for dynamic and reconfigurable crosslinking to modulate functionality. In this study, the *Hydra magnipapillata* minicollagen-1 isoform was recombinantly expressed in bacteria, and a high throughput purification protocol was developed to generate milligram levels of pure protein without column chromatography. The resulting minicollagen-1 preparation demonstrated spectral properties similar to those observed with collagen and polyproline sequences as well as the ability to self-assemble into oriented fibers and bundles. Photo-crosslinking with Ru(II)${{\left(\text{bpy}\right)}_{3}}^{2+}$ was used to create robust hydrogels that were analyzed by mechanical testing. Interestingly, the minicollagen-1 hydrogels could be dissolved with reducing agents, indicating that ruthenium-mediated photo-crosslinking was able to induce disulfide metathesis to create the hydrogels. Together, this work is an important first step in creating minicollagen-based materials whose properties can be manipulated through static and reconfigurable post-translational modifications.

## Introduction

Marine organisms have proven to be a rich resource for the discovery of biomaterials with unique properties that hold promise for the creation of new advanced materials, such as strong, lightweight polymers, adhesives and biophotonic structures (Crookes et al., 2004; Degtyar et al., 2014; Petrone et al., 2015). The phylum *Cnidaria* includes sea anemones, corals as well as jellyfish, and members of this phylum possess specialized stinging cells, termed nematocysts, used for prey capture, defense and locomotion (Ozbek et al., 2009). Upon stimulation, the nematocyst undergoes explosive release of a stylet within 700 ns, generating an acceleration of 5,400,000 x g (Nuchter et al., 2006). The rapid deployment of the piercing structure is enabled due to the high osmotic pressure (150 bars) stored in the nematocyst capsule (Weber, 1989), which is generated by the accumulation of polyglutamic acid and ions within the capsule (Weber, 1990). In addition to the stylet, a tubule is everted from the nematocyst for the delivery of venom (Ozbek et al., 2009). The nematocyst capsule is composed of outer and inner walls, where the function of the outer wall is proposed to direct organization and assembly of the inner wall (Engel et al., 2002). It has been demonstrated by specific proteolytic digestion of the outer wall that the inner wall is sufficient for the containment of the high nematocyst osmotic pressure (Kurz et al., 1991). High resolution microscopy showed that the inner wall is made up of oriented fibrils that display a striated pattern reminiscent of banding patterns observed with collagen fibers (Holstein et al., 1994). Screening of a *Hydra magnipapillata* capsule wall cDNA library indicated the presence of small molecular weight structural proteins, termed minicollagens, consisting of a central domain made up of collagen-like Gly-X-Y repeats, where the X and Y positions are high in proline (Kurz et al., 1991). Flanking the central collagen-like domain are two proline-rich domains that are in turn flanked by cysteine-rich domains (CRDs) at the amino and carboxy termini of the protein (Kurz et al., 1991). Like long collagens, minicollagens form a complex made up of three polypeptide chains where the polyproline regions and terminal CRDs are thought to splay out from a rigid, trihelical rod-like collagen center (Engel et al., 2001). In *H. magnipapillata*, 21 minicollagen isoforms were identified through proteomic analysis, and members of the minicollagen family were found as components of the inner capsule wall as well as the venom delivering tubule (Kurz et al., 1991; Engel et al., 2001; Adamczyk et al., 2008; Balasubramanian et al., 2012). The key domains involved in assembly of nematocyst structures are thought to be the CRDs, which have been identified in other nematocyst proteins in addition to the minicollagens (Kurz et al., 1991; Engel et al., 2002; Balasubramanian et al., 2012). The CRDs are proposed to perform two functions in the creation of nematocyst structures. First, they function as multimerization domains that control organization and self-assembly of higher order protein complexes (Meier et al., 2007; Tursch et al., 2016). Second, they contain stored intrachain disulfide bonds that are mobilized at the right time to create interchain crosslinks that stabilize the protein complexes (Meier et al., 2007; Beckmann et al., 2015). Thus, it is proposed that compaction and hardening of minicollagen oriented fibrils occurs through disulfide bond isomerization of the cysteines contained in the terminal cysteine rich domains (Engel et al., 2001). In this model, minicollagens represent a “brick-and-mortar” approach to the creation of strong, bio-based materials, where individual multimeric units can be patterned and enzymatically or chemically crosslinked into tough, resilient final materials.

Such a material could be leveraged as a protein-based hydrogel, a class of material that has found extensive use in tissue engineering due to inherent biocompatibility (Zhang and Khademhosseini, 2017). Collagen-based hydrogels (particularly Type I) are popular substrates for cell culturing and tissue engineering due to the abundance of collagen in natural extracellular matrices (Antoine et al., 2014, 2015). In recent years, to enhance hydrogel tunability and mechanical strength, collagen has been functionalized or additional crosslinkers have been added which compromise the biocompatibility of the resulting hydrogels (Antoine et al., 2015; Li et al., 2018). One simple method to create tunable protein-based hydrogels has been established via controlling the level of disulfide crosslinking in keratin-based gels where intramolecular disulfide bonds from cysteine side chains were reduced and later oxidized to forms intermolecular disulfide bonds to construct keratin-based hydrogels (Cao et al., 2019). Minicollagen-1 as a potential material for protein-based hydrogels may possess both the biocompatibility of collagen and tunability of keratin by disulfide crosslinking of its cysteine rich domains.

In this study, the *H. magnipapillata* minicollagen isoform, minicollagen-1, is expressed and purified from an *E. coli* expression system. Secondary structure analysis is carried out to determine how the recombinant minicollagen isoform compares to collagen and polyproline sequences in response to environmental perturbations. Electron microscopy is also employed to study how self-assembly of the minicollagen from the prokaryotic expression system compares to minicollagen preparations synthesized in mammalian cell culture systems. Finally, taking advantage of the relatively high levels of minicollagen afforded by bacterial expression, hydrogels are created and studied for their mechanical properties.

## Materials and Methods

All reagents were obtained from Sigma-Aldrich and were used as received, unless otherwise mentioned. All solutions were made using water with resistivity recorded at 18.2 MΩ·cm obtained from a Barnstead GenPure system. Corning pH meter 430 was used to adjust the pH of stirred solutions by dropwise addition of either acid or base as required.

### Expression, Purification, and Staining of Recombinant recMinicollagen-1 in *Escherichia coli*

A codon optimized minicollagen-1 isoform coding sequence was synthesized (GenScript, Piscataway, NJ) and cloned into a pST44 expression vector (Tan et al., 2005) under ampicillin selection. The resulting pST44-minicollagen-1 or pST44-EFP-minicollagen-1 construct was then transformed into *E. coli* BL21 DE3 cells. Four liters of autoclaved LB media was inoculated and cells were grown at 37°C using with continuous air sparging of the media. Once cell cultures reached an optical density (OD_600nm_) of 0.6–0.8, expression of the minicollagen-1 was induced with 0.4 mM isopropyl β-D-1-thiogalactopyranoside (IPTG obtained from GOLDBio) for 18 h at 25°C. Bacterial cultures were then centrifuged and then re-suspended with lysis buffer (50 mM Tris pH 8, 100 mM NaCl, 100 μg/mL lysozyme) followed by sonication. Lysed cells were then heat treated at 80°C, for 30 min followed by cooling in an ice bath for another 30 min. Denatured contaminant proteins were removed by centrifugation and the supernatant was adjusted to a pH of 3.5 using 1M HCl followed by centrifugation. The supernatant was then adjusted to pH of 8.5 with 1M NaOH, followed by lyophilization to dryness. The resulting protein powder was dissolved in 25 ml of deionized water and treated with equal volume of saturated ammonium sulfate with continuous stirring. The precipitate was removed by centrifugation and re-suspended in 10 mL of deionized water. Ten milliliters of absolute ethanol was then added and the sample cooled at −20°C for 1 h before centrifugation. The supernatant was mixed with 10 mL of saturated ammonium sulfate and shaken vigorously followed by centrifugation in a swinging bucket rotor. The top organic phase containing the expressed minicollagen-1 was then removed and evaporated to dryness. The powder was re-suspended in 10 mL deionized water followed by dialysis in deionized water 24 h. The minicollagen-1 sample was then lyophilized to dryness, weighed and the purified protein powder stored at −80°C until use.

Minicollagen-1 samples were run on 12% polyacrylamide gel and fixed in 20% glacial acetic acid and 15% methanol for 30 min. The gels were then stained in solution of 20% glacial acetic acid, 15% methanol and 0.1% Coomassie R-250 for 24 h at room temperature.

### Circular Dichroism Spectra of Recombinant Minicollagen-1

Circular dichroism spectra were collected on a Jasco J-815 CD spectrometer from 300 to 180 nm at a scan rate of 20 nm/min and averaged over 3 repetitive scans using a quartz cuvette with a 1 cm pathlength. Minicollagen-1 was prepared at a concentration of 1.4 μM in water and treated with select agents. CD spectra were collected and subtracted from the corresponding reference spectrum of water, or the treatment agent only. Temperature studies were performed using a peltier controlled cuvette holder. CD spectra were collected for minicollagen-1 during heating at 25°C, 50°C, 70°C, and 90°C.

### Transmission Electron Microscopy of Recombinant Minicollagen-1

To prepare samples for TEM, 4 μl of either undiluted (1 mg/ml) or diluted (0.1 mg/ml) minicollagen-1 was applied to a 400 mesh copper grid supporting a thin carbon film. The sample was allowed to adhere for 1 min before staining with Uranyl-less stain for 3 min, washing, then blotting to dryness with Whatman #1 filter paper. Samples were imaged on a Ceta camera on a Thermo Fisher Scientific Talos TEM operating at 200 kV and using a 40 μm objective aperture.

### Ellman's Reagent Assay

Quantification of free sulfhydryl on bacterially expressed minicollagen-1 protein was obtained by using cysteine hydrochloride monohydrate as a standard for the Ellman's reagent assay (ThermoScientific). The reaction buffer contains 0.1 M sodium phosphate buffer solution (pH 8.0), and 1 mM ethylenediaminetetraacetic acid (EDTA). The reagent solution were prepared by dissolving 4 mg of Ellman's reagent in 1 ml of reaction buffer. Various concentrations of standard was prepared by dissolving cysteine-HCl in reaction buffer. Minicollagen-1 protein then dissolved in reaction buffer to final concentration of 1 mg/ml. For the assay, 15 μl of each sample was mixed with 150 μl of Ellman's reagent solution and incubated at room temperature for 15 min. Then the absorbance at 412 nm was obtained using the plate reader (SpectraMax M3, Molecular Device).

### Photo-Crosslinking of Recombinant Minicollagen-1 Hydrogels

Solutions of minicollagen-1 in water were photo-chemically crosslinked to form hydrogels using tris(2,2-bipyridyl) dichloro ruthenium (II) hexahydrate (Ru(II)${{\left(\text{bpy}\right)}_{3}}^{2+}$) as the catalyst and ammonium persulphate (Bio Rad) as the electron acceptor. Hydrogel cylinders for mechanical testing were prepared from a 17 wt% minicollagen-1 solution containing 37 mM ammonium persulfate and 2.2 mM Ru(II)${{\left(\text{bpy}\right)}_{3}}^{2+}$. The mixture was transferred into cylinders cut out of a small transfer pipette bulb (1.5 ml) and photo-crosslinked under white lamp light within 5 min. Post crosslinking, the hydrogels were transferred into 20 mM EDTA and 10 mM EGTA for removal of the photo-crosslinker catalyst. The crosslinked cylinders are 2.5 mm in diameter and 3–4 mm in height. To evaluate effect of reducing agent on the hydrogels, 10 wt% of rhodamine labeled minicollagen hydrogel was made at the bottom of an Eppendorf tube cap (dome shaped hydrogel) using the above photo-crosslinking protocol. Hydrogels were then submerged in solutions of DTT (10 mM), CRB (10 mM EDTA, 1 mM EGTA, 1% β-mercaptoethanol), βME (1% β-mercaptoethanol) and 10 mM EDTA/1 mM EGTA for 3 h to analyze the effect of the reducing reagents.

### Determination of Equilibrium Swelling Degree

The swelling capacity of minicollagen-1 hydrogels was determined by incubating them in deionized water at room temperature for 2 days and then drying the hydrogels at room temperature until no change in dimensions of hydrogel was observed. The swelling degree was calculated using the following equation:

$$\begin{array}{l} Swelling\;degree=\frac{Vs-Vd}{Vd}\;\times 100 \end{array}$$

Where *Vs* and *Vd* are, respectively, volume of hydrogel in swollen and dried state.

### Mechanical Testing

Compressive modulus of 17 wt% minicollagen-1 hydrogel was performed using CellScale Microsquisher (Microtester G2). In this setup, a tungsten microbeam of 0.5588 mm in diameter is fixed on one end to a vertical actuator and on the other end to a 4 × 4 mm^2^ compression platen, which was tracked by a camera to record the displacement. As the beam moved to compress the hydrogel, deflection was measured in real time by the relative position of the beam at the camera and at the motor. Force was calculated by the Microsquisher software using the following equation:

$$\begin{array}{l} Force=\frac{Deflection\times 3\times Beam\;Modulus\times Beam\;Area\;Moment\;of\;Intertia\;}{{Beam\;Length}^{3}\;} \end{array}$$

Where

$$\begin{array}{l} Beam\;Area\;Moment\;of\;Intertia=\frac{\pi {\times radius}^{4}}{4} \end{array}$$

Each gel was sequentially compressed to final strains of 5, 10, and 15% within 50 s, held for 1 s followed by recovery within 50 s. The compressive elastic modulus was calculated as the slope of the linear portion of the stress versus strain curve and reported for each final strain as an average of measurements obtained from three different gels.

## Results

**Figure 1:**
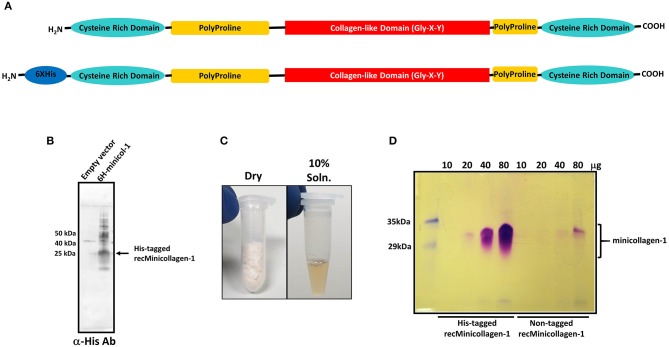
**(A)** Schematic of the minicollagen-1 domain structure with the individual domains labeled. **(B)** Western blot analysis of extracts from bacteria transformed with empty vector or a vector containing the hexa-histidine tagged minicollagen-1. The blot was probed with an anti-hexa-histidine antibody and the position of the tagged recombinant minicollagen-1 is indicated. **(C)** Purified, lyophilized recombinant minicollagen-1 (30 mg) is shown (left panel) with a 10 wt% solution of recombinant minicollagen-1 in water (right panel). **(D)** SDS-PAGE of the hexa-histidine-tagged and non-tagged recombinant minicollagen-1 expressed and purified from bacteria after staining with Coomassie R-250. The amount of protein loaded in each lane is indicated at the top.

Previous studies have identified the minicollagens as a major component of the nematocyst capsule inner wall (Kurz et al., 1991; Holstein et al., 1994). Up to now, there have been no studies of minicollagen mechanical properties from hydrogels made of the isolated protein. In order to determine how a crosslinked minicollagen network may function to form an elastic material capable of withstanding extreme pressures, we have synthesized the coding region for minicollagen-1 from *H. magnipapillata* and subcloned it into a bacterial expression vector for large scale production in *E. coli*. The *H. magnipapillata* minicollagen-1 has been well-studied and represents the canonical form of the minicollagen family with a central collagen-like set of 14 Gly-X-Y repeats immediately flanked at both ends with polyproline regions and cysteine rich domains at both the amino and carboxyl termini (Figure 1A). Thus, proline is the most abundant amino acid in minicollagen primary structures. In minicollagen-1, prolines occupy the Y position of 86% of the three amino acid repeats and at both the X and Y positions in 36% of the repeats (Supplementary Figure 1). Both hexa-histidine tagged and untagged versions of minicollagen-1 were created for bacterial expression in *E. coli*, where the hexa-histidine tagged form of minicollagen-1 was initially used for screening of bacterial expression. Western blotting of His-minicollagen-1 isolated using IMAC yielded a dominant polypeptide with a 25–30 kDa apparent molecular weight that cross reacted with an anti-hexa-histidine antibody (Figure 1B). When expression was carried out at a larger scale, the net yields of recombinant minicollagen-1 were poor. This was likely due to the consecutive proline stretches before and after the collagen-like domain that are known to cause translational stalling in other proteins (Doerfel et al., 2013; Ude et al., 2013). To mitigate this, a bicistronic construct was created where the *E. coli* translation elongation factor EF-P was expressed with the His-tagged minicollagen-1 (Supplementary Figure 2). EF-P was previously reported to increase expression of proteins that contain stretches of more than two consecutive prolines by limiting ribosomal stalling that occurs when a third consecutive proline is being translated (Doerfel et al., 2013; Ude et al., 2013). One observation that assisted in the identification of purified, recombinant minicollagen-1 by SDS-PAGE was that it stained metachromatically with Coomassie R-250, a phenomenon observed with mammalian collagens and other proteins with high proline content (Hattori et al., 1996). Metachromatic staining results in purple rather than the blue staining observed with most other orthochromatically stained proteins and gives strong support to the conclusion that the purified material is minicollagen-1 (Supplementary Figure 2A). *E. coli* was transformed with minicollagen-1 constructs either without or with co-expressed EF-P and eight colonies of each were analyzed for minicollagen-1 expression by Coomassie R-250 metachromatic staining (Supplementary Figures 2B,C). The results indicated that co-expression with EF-P did result in increased expression of minicollagen-1 by ~3 fold (Supplementary Figures 2B,C). Therefore, all future large scale expressions of recombinant minicollagen-1 were carried out with co-expressed EF-P.

Although the hexa-histidine tag would allow for column purification of the recombinant minicollagen-1, a purification protocol was developed where the tag would not be needed so that non-tagged versions of recombinant minicollagen-1 could also be purified in large amounts without column chromatography (Materials and Methods). The final step in the optimized minicollagen purification protocol is lyophilization which produces an off white, cotton-like protein sample that demonstrates good solubility in water, easily dissolving at concentrations up to 10 wt% (Figure 1C). As was observed with the initial Western blotting of the recombinant minicollagen-1, SDS-PAGE analysis of the purified His-tagged and non-tagged variants, followed by Coomassie R-250 staining indicated an abnormally high apparent molecular weight for the monomeric form of the protein (between 30 and 35 kDa) that stained metachromatically (Figure 1D). This apparent molecular weight is likely due to the particular characteristics of the minicollagen-1 protein in terms of its ability to bind SDS, as mass spectrometry analysis indicated a peak with good agreement to the predicted mass of the monomeric protein (Supplementary Figure 3). The lack of additional orthochromatically stained proteins on the gel, even at high levels of protein loading, indicated that the recombinant minicollagen-1 preparations were largely free of contaminating proteins. The net yield of purified, recombinant minicollagen-1 was estimated at ~18 mg per liter of culture. One additional characteristic that makes analysis of recombinant minicollagen-1 expression difficult using SDS-PAGE is its tendency to diffuse out of the gel during fixing and staining. This greatly reduces the ability to see low levels of protein using SDS-PAGE, however the presence of a hexa-histidine tag decreases the diffusion of recombinant minicollagen-1 out of the gel compared to the non-tagged version of the protein (Figure 1D). Together the data indicate that *E. coli* is able to heterologously express Hydra minicollagen-1 in milligram quantities. Furthermore, we have developed an easily scalable columnless purification protocol that yields recombinant minicollagen-1 with near homogenous protein purity.

**Figure 2:**
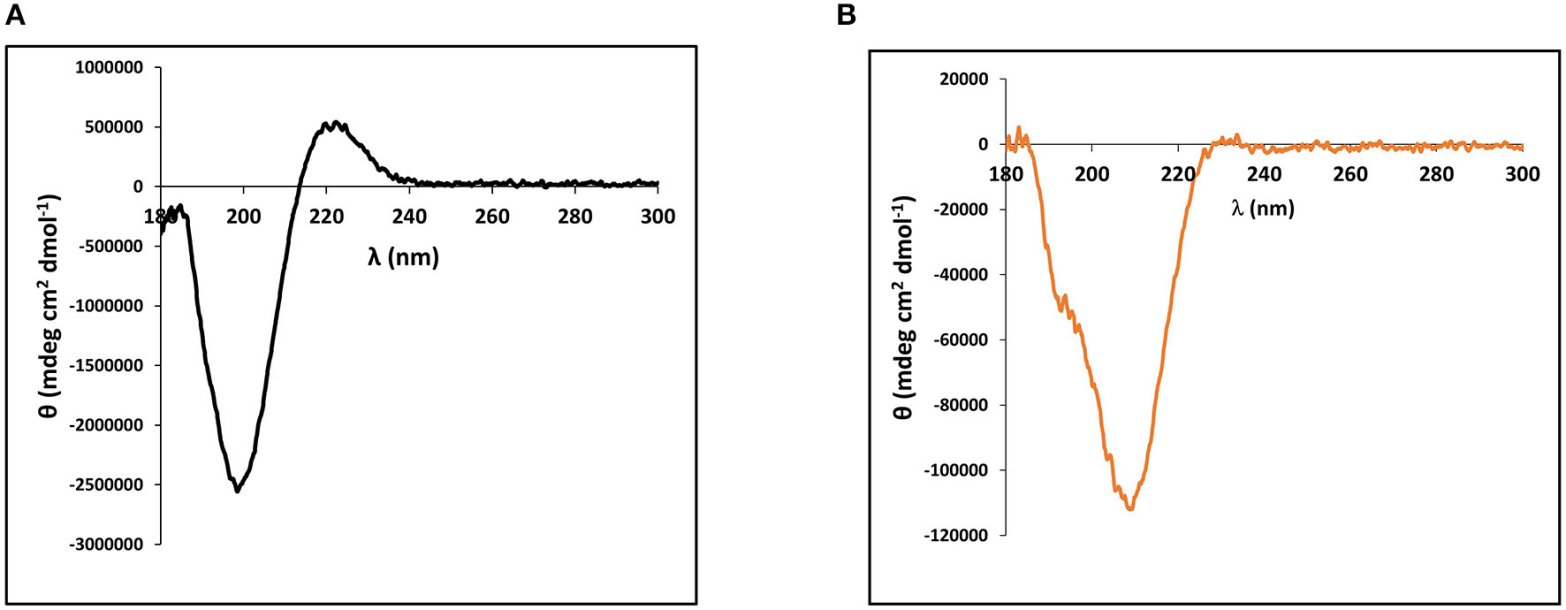
Circular dichroism (CD) spectroscopy performed on **(A)** 90 nM type I bovine dermal collagen and **(B)** Solution of 1.4 μM of purified, bacterially expressed, non-tagged recombinant minicollagen-1.

The cnidarian minicollagens are unique in that they contain both G-X-Y collagen-like repeats as well as flanking polyproline stretches. Many studies have looked into secondary structure characterization of both of these proline-rich sequences individually (Tiffany and Krimm, 1972; Gutierrez-Cruz et al., 2001; Whittington et al., 2005; Lopes et al., 2014). Thus, secondary structure of the purified, recombinant minicollagen-1 was compared to the secondary structure of a high molecular weight collagen. For this, circular dichroism (CD) spectroscopy was performed on the recombinant minicollagen-1 and purified type I bovine dermal collagen. The CD spectrum for type I bovine collagen demonstrated a strong negative ellipticity at 198 nm and a pronounced positive ellipticity at 222 nm (Figure 2A). These absorbances are hallmarks of the extended polyproline II helix found in the trihelical quaternary structure of the long collagens and are in good agreement with numerous other collagen CD spectra published previously (Tiffany and Krimm, 1972; Lopes et al., 2014). CD spectroscopy of the recombinant minicollagen-1 demonstrated a clear negative ellipticity at 207 nm, with a small hump often observed below 200 nm, and a minor positive ellipticity seen at 222 nm similar to type I bovine collagen (Figure 2B). The presence of the hexa-histidine tag had little effect on the recombinant minicollagen-1 spectrum, with the spectra for the two recombinant minicollagen-1 forms being nearly identical (Supplementary Figure 4). The CD spectra for both forms of the recombinant minicollagen-1 are similar to those observed for polyproline sequences which adopt an extended polyproline II secondary structure (Tiffany and Krimm, 1972; Gutierrez-Cruz et al., 2001; Whittington et al., 2005; Lopes et al., 2014). However, it does not appear that the recombinant minicollageni is adopting a trihelical quaternary structure (see Discussion).

**Figure 3:**
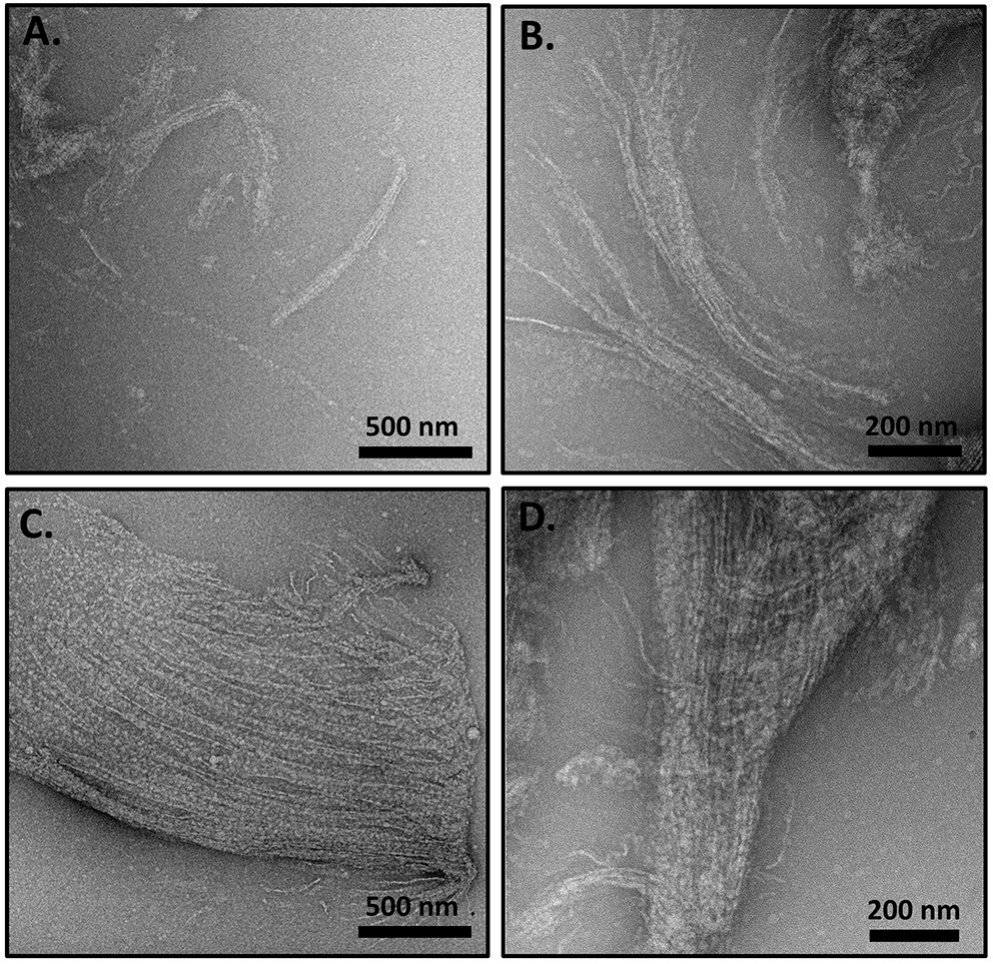
**(A–D)** TEM analysis of concentrated (1 mg/ml), non-tagged recombinant minicollagen-1 purified from bacteria. The scale bar distance is indicated on the figure.

TEM analysis of the nematocyst inner capsule wall indicates an ordered array of striated structures that are reminiscent of ordering observed with the long collagens (Starborg et al., 2013). Additionally, minicollagen-1 expressed in a human cell line forms rod like structures that self-organize into oriented bundles (Engel et al., 2001). Therefore, the ability of the recombinant minicollagen-1 to self-assemble into ordered structures was analyzed by TEM. A dilute solution (0.1 mg/ml) of the non-tagged recombinant minicollagen-1 was dropped cast onto a TEM grid and analyzed after staining (Supplementary Figure 5). The resulting micrographs showed a predominance of small lightly stained globular structures throughout the visual field with no indication of higher ordered structuring. When the non-tagged recombinant minicollagen-1 sample was concentrated 10 fold (1 mg/ml) and the TEM analysis repeated, significant self-assembly was observed (Figure 3). Bundles of fibrils could be seen with various levels of organization. In some fields, small fibril bundles were observed with chains of globules (Figure 3A). In other fields, fibrils were seen coming together to form small (Figure 3B) or large (Figure 3C) bundles where the fibrils appear both globular and defined in certain areas of the bundle. Some oriented structures were observed to be very compact, demonstrating a striated appearance that indicates a tighter packing of the fibrils (Figure 3D). When a high concentration of the hexa-histidine tagged version of minicollagen-1 was analyzed by TEM, self-assembly appeared to occur, but the resulting structures were less oriented demonstrating a more chaotic organization (Supplementary Figure 6). This lack of oriented self-assembly may explain why the hexa-histidine tagged form of recombinant minicollagen-1 fixes well in the gel after SDS-PAGE compared to the non-tagged version of the protein. Together, the data suggest that recombinant minicollagen-1 self assembles into highly oriented fiber bundles in response to increasing protein concentration and that this ordering is disrupted with hexa-histidine modification of the amino terminus.

**Figure 4:**
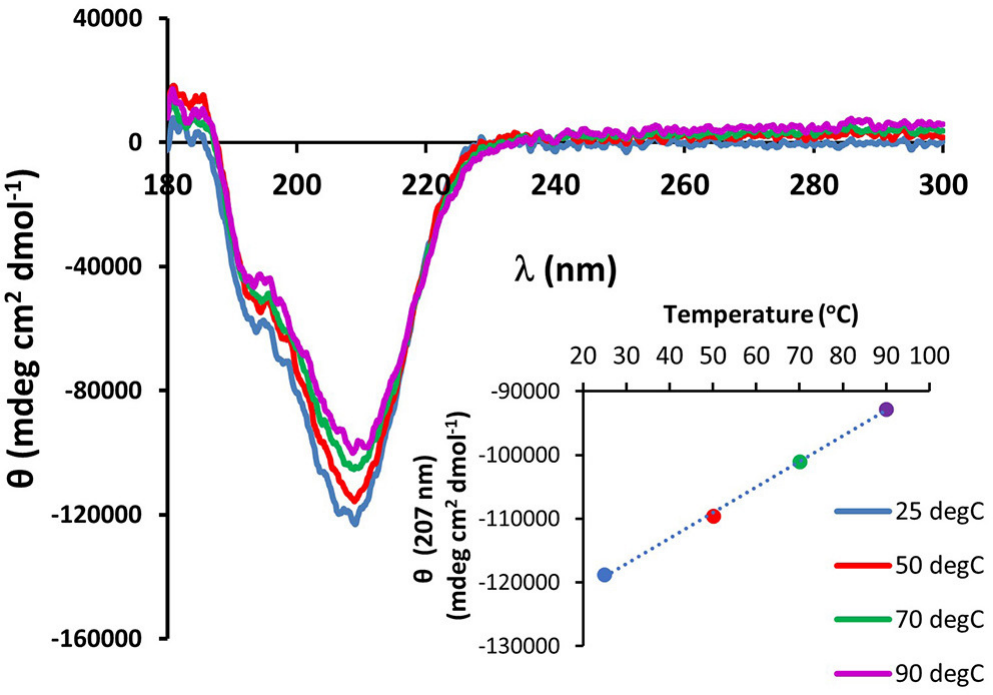
CD spectroscopy of non-tagged recombinant minicollagen-1 (1.4 μM) with increasing temperatures. The temperatures are indicated on the figure. The ellipticity measured at 207 nm as a function of temperature is plotted (inset).

The existence of the G-X-Y collagen-like repeats and the stretches of polyproline present in the minicollagen-1 primary structure raised the question of how similar the properties of recombinant minicollagen-1 were to those of polyproline sequences and the high molecular weight collagens. The sensitivity of the long collagens to temperature is well documented (Tiffany and Krimm, 1972; Lopes et al., 2014), therefore the thermal stability of the recombinant minicollagen-1 secondary structure was measured by CD spectroscopy while exposed to increasing temperatures ranging from 25 to 90°C (Figure 4). The secondary structure of recombinant minicollagen-1 demonstrated remarkable thermal stability as evidenced by minor changes in the negative elipticity at 207 nm as well as the minor hump below 200 nm. These ellipticities decreased somewhat as the temperature increased from 25°C to 90°C. Heating the recombinant minicollagen-1 solution to 70°C showed a 20% decrease of the negative ellipticity observed at 207 nm (Figure 4, inset), whereas mammalian collagen has been reported to undergo significant denaturation at higher temperatures (Tiffany and Krimm, 1972). The thermal stability of recombinant minicollagen-1 is a useful tool in its purification from soluble bacterial extracts, as it remains in solution after heat treatment of the extracts at 80°C, while a majority of the bacterial contaminating proteins precipitate. The CD spectrum of recombinant minicollagen-1 was also measured in the presence of 1M urea, a chaotrope that has been shown to significantly affect the secondary structure of collagen (Lopes et al., 2014). In contrast to the thermal stability observed earlier, the recombinant minicollagen-1 CD spectrum demonstrated significant changes in the presence of urea, with the negative ellipticity at 207 nm being completely abrogated in the presence of the chaotrope (Supplementary Figure 7). The only signature peak remaining in the spectrum displayed a small negative ellipticity at 218 nm. In total, this indicates an increase in random conformational states, and potential urea resistant aggregation of recombinant minicollagen-1.

**Figure 5:**
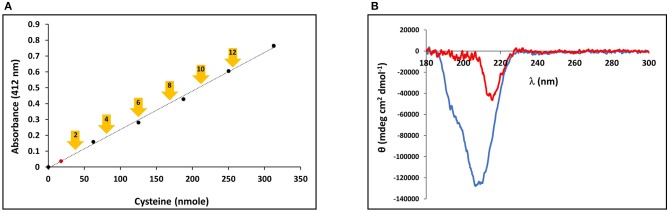
**(A)** Calibration curve of Ellman's reagent reduction with the assayed amounts of reduced cysteine indicated. The measured absorbance of 21.5 nmoles minicollagen-1 assayed with Ellman's reagent is indicated as a red dot on the calibration curve. The theoretical absorbance expected for Ellman's reagent reduction by the indicated number of recombinant minicollagen-1 free sulfhydryls based on the 21.5 nmoles of the recombinant minicollagen-1 assayed (yellow arrows). **(B)** CD spectroscopy of non-tagged recombinant minicollagen-1 (1.4 μM) in the absence (blue line) and presence (red line) of 1 mM DTT.

The presence of the CRDs at the amino and carboxy termini of the minicollagens distinguishes them from the other collagens and suggests that their structure may be modulated by the redox state of the sulfhydryl side chains. In order to measure the oxidation state of the cysteine sulfurs in the purified, recombinant minicollagen-1, a colorimetric assay was performed using Ellman's reagent, where a chromophore is produced upon reduction of the reagent's disulfide bonds in the presence of reducing free sulfhydryl groups from an analyte (Ellman, 1959). For comparison, a calibration plot was created using reduced cysteine to indicate the absorbance generated from Ellman's reagent per reducing equivalent present in the reaction (Figure 5A). There are 12 cysteines present per minicollagen-1 monomer, so the theoretical positions of all potential reducing equivalents (in groups of two) from the assayed 21.5 nmoles of recombinant minicollagen-1 were indicated on the calibration plot (Figure 5A). When the recombinant minicollagen-1 (21.5 nmoles) was reacted with Ellman's reagent, the resulting absorbance was less than the theoretical value for the presence of one reducing equivalent, indicating that all of the cysteine sulfurs in the recombinant minicollagen-1 were oxidized as disulfides. This is consistent with the idea that minicollagens store disulfide bonds intra-molecularly to be mobilized for the creation of mechanically robust materials (Engel et al., 2001). To test the role of the disulfide bonds in the structure of recombinant minicollagen-1, its CD spectrum was determined in the presence of 1 mM DTT. Similar to the results obtained with urea, the presence of DTT strongly decreased the negative elipticity observed at 207 nm, while leaving a small negative ellipticity at 218 nm (Figure 5B). In contrast, long collagen structure, as determined by CD spectroscopy, has been shown to be unaffected by DTT concentrations up to 5 mM (Lopes et al., 2014). Furthermore, the reduction of recombinant minicollagen-1 leads to increased migration on SDS-PAGE, suggesting that disulfide bond reduction alters the conformation of the protein (Supplementary Figure 8). The data indicate that disulfide bonding between sulfhydryls in the cysteine rich domains is a significant factor in stabilizing the secondary structure of recombinant minicollagen-1, consistent with the proposed role of these domains in regulating the material properties of the inner nematocyst wall.

**Figure 6:**
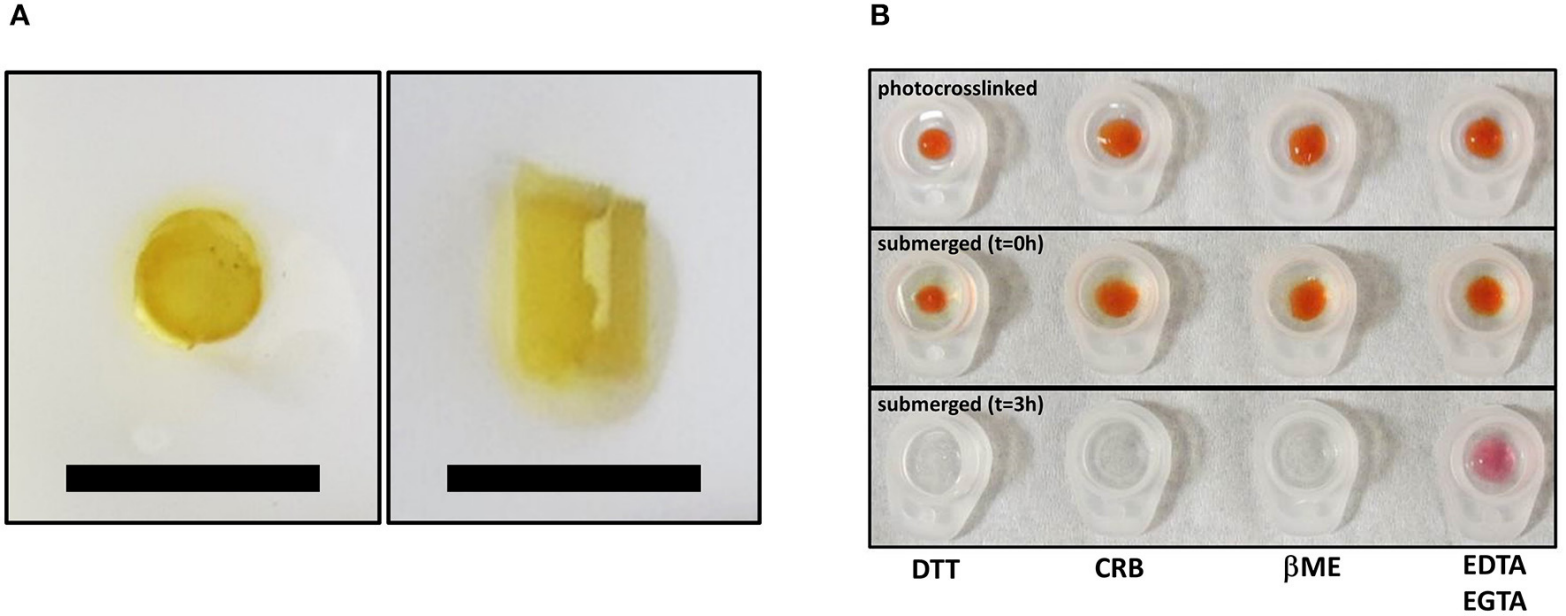
**(A)** Top view (left) and side view (right) of same free-standing, photo-crosslinked non-tagged recombinant minicollagen-1 (17 wt%) hydrogels that was used for mechanical testing. The scale bars are 1 cm. **(B)** Photo-crosslinked dome shaped hydrogels of 10 wt% minicollagen (doped with rhodamine labeled recombinant minicollagen-1 before removal of ${\text{Ru}(\text{II})(\text{bpy})}_{3}^{2+}$ (upper) and immediately after submersion in the indicated treatment solutions (*t* = 0 h), or after 3 h in the indicated solutions. DTT (10 mM), CRB (10 mM EDTA, 1 mM EGTA, 1% β-mercaptoethanol), βME (1% β-mercaptoethanol), and 10 mM EDTA/1 mM EGTA are indicated on the figure.

**Figure 7:**
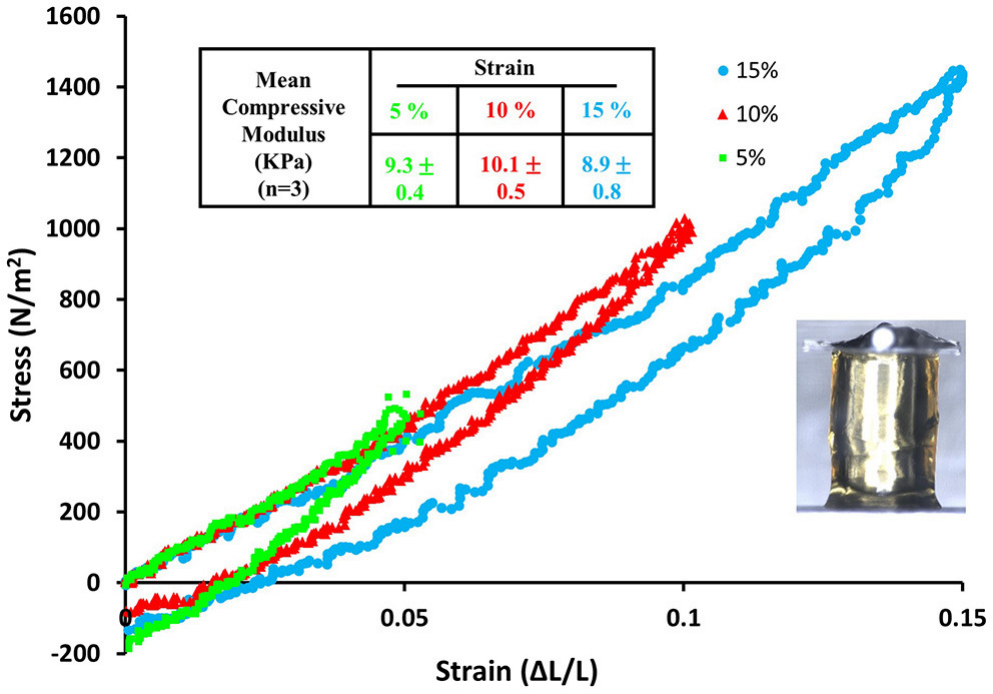
Stress vs. strain curve for compression and then recovery of a 17 wt% recombinant minicollagen-1 photo-crosslinked hydrogel obtained by sequential 5, 10, and 15 % compression. The table on top of the graph summarizes the mean compressive modulus that was calculated from mechanical testing of three hydrogels. The inset image shows the recombinant minicollagen hydrogel under compression using the Microtester G2.

Mobilization of minicollagen cysteine disulfide bonds is proposed to be a key mechanism in the formation and sclerotization of the nematocyst inner wall (Engel et al., 2001). To date, the natural mechanism of disulfide metathesis in the nematocyst inner wall is unknown, so an artificial stimulus to induce disulfide bond rearrangement was sought in order to create recombinant minicollagen-1 hydrogels. The photo-catalyst ${\text{Ru}(\text{II})(\text{bpy})}_{3}^{2+}$ can create protein hydrogels through sulfate radical chemistry and the formation of dityrosine crosslinks (Fancy and Kodadek, 1999; Elvin et al., 2005). Other studies have utilized sulfate radical chemistry to create hydrogels crosslinked with disulfide bonds that are degradable with reducing agents (Gaulding et al., 2012). Therefore, ${\text{Ru}(\text{II})(\text{bpy})}_{3}^{2+}$ mediated photo-crosslinking was tested for its ability to initiate disulfide metathesis and inter-chain crosslinking of recombinant minicollagen-1 to create hydrogels. By photo crosslinking (as described in Materials and Methods), the recombinant minicollagen-1 solution demonstrated clear gelation and could be cast into different shapes (cylindrical shape for mechanical testing and dome shape for evaluating effect of reducing reagents) as well as immersed in aqueous salt solutions without dissolving (Figure 6). To test the stability of the hydrogels in the presence of a reducing environment, small 20 μl recombinant minicollagen-1 hydrogels were created with a small amount of rhodamine labeled recombinant minicollagen-1 added to more easily visualize the resulting hydrogels once the photo-initiator was removed. After chelation of the ${\text{Ru}(\text{II})(\text{bpy})}_{3}^{2+}$, a water stable pink hydrogel was observed (Figure 6B, lower panel). However, when a reducing agent (β-mercaptoethanol) was added to the chelation mix (CRB), the recombinant minicollagen-1 hydrogels dissolved (Figure 6B). The dissolution of photo-crosslinked recombinant minicollagen-1 hydrogels was also observed when β-mercaptoethanol was added alone or when a different reducing agent (DTT) was added. Thus, ${\text{Ru}(\text{II})(\text{bpy})}_{3}^{2+}$ mediated photo-crosslinking can create hydrogels of recombinant minicollagen-1, at least in part, through the metathesis of disulfide bonds present in the terminal cysteine rich domains. In order to measure mechanical properties, cylindrical hydrogels were cast from 17 wt% aqueous solution of minicollagen-1 as described (Materials and Methods). The equilibrium swelling degree of 17 wt% minicollagen-1 hydrogel was calculated as 5.24 ± 0.85 in deionized water. The compressive elastic modulus was measured using a CellScale Microtester G2 (Figure 7). The minicollagen-1 hydrogels were found to have nearly linear stress response up to the maximum measured strain of 15% and to unload with minimal hysteresis (Figure 7). The compressive elastic modulus was found to be 9.3 ± 0.4 KPa. Previously, it was shown that the marine sandworm protein, Nvjp-1, could be made into hydrogels through photo-crosslinking with ${\text{Ru}(\text{II})(\text{bpy})}_{3}^{2+}$ or crosslinking with horseradish peroxidase (Chou et al., 2017; Gupta et al., 2018). Nvjp-1 hydrogel creation with these reagents occurs through the formation of dityrosine crosslinks (Fancy and Kodadek, 1999; Partlow et al., 2014). The compressive elastic modulus of photo-crosslinked minicollagen-1 hydrogels compared favorably with that of horseradish peroxidase catalyzed Nvjp-1 hydrogels (Supplementary Figure 9). These data show that minicollagen-1 crosslinking through disulfide metathesis is able to create robust hydrogels whose mechanical properties are comparable to protein hydrogels created with irreversible covalent crosslinks.

## Discussion

In this study, the heterologous expression and purification of recombinant minicollagen-1 in *E. coli* at milligram levels is demonstrated for the first time, and the ability to express and purify relatively large amount of the protein has enabled the creation of hydrogels that can be mechanically tested. However, there are difficulties that heterologous expression of recombinant minicollagen-1 presents, and yields will need to be increased if recombinant minicollagens are to be used for practical applications. The culture conditions were important in increasing the yield of minicollagen-1, where induction at low temperatures (25°C) and constant oxygenation through air sparging were found to increase expression levels. The low net yields of minicollagen-1 may be due to losses experienced during purification, but are also likely due to poor expression resulting from ribosomal stalling at the long stretches of prolines that flank the central collagen-like domain. Earlier studies showed that the bacterial translation elongation factor, EF-P, is responsible for mediating expression of transcripts coding for stretches of proline longer than two residues (Doerfel et al., 2013; Ude et al., 2013). A bicistronic construct co-expressing EF-P with the His-tagged recombinant minicollagen-1 was created and tested for His-recombinant minicollagen-1 expression. Although co-expression of EF-P with recombinant minicollagen-1 increased expression levels, it is possible that expression can be further increased. EF-P is post-translationally modified with a β-lysyllysine that is essential for function (Park et al., 2012). Thus, it is possible that the over-expressed EF-P is sub-stoichiometrically modified and therefore not operating at full functionality. The gene product YjeK is responsible for the isomerization reaction that creates the β-lysine from α-lysine, and YjeA is the ligase that attaches the β-lysine moiety to a lysine side chain on EF-P (Yanagisawa et al., 2010). Future attempts at expression optimization will create a polycistronic construct where the EF-P modifying enzymes, YjeK and YjeA, will be co-expressed with the EF-P and recombinant minicollagen-1. This approach will hopefully create a system whereby proteins and peptides with high proline content can be efficiently produced in a prokaryotic system.

A key question related to recombinant minicollagen-1 is whether it forms the trimeric quaternary structure observed for the long collagens as well as minicollagens heterologously expressed in mammalian cell culture platforms (Engel et al., 2001). Proline hydroxylation is a post-translational modification that has been implicated in the stabilization of the extended collagen triple helix, and previous studies indicate that the minicollagens also contain hydroxyproline (Lenhoff et al., 1957; Engel et al., 2001). The heterologously expressed recombinant minicollagen-1 is expected to contain no proline hydroxylation, as is known for the bacterial collagens (Mohs et al., 2007). This combined with the short collagen-like region contained in recombinant minicollagen-1 suggests that formation of the triple helical quaternary structure would be unfavorable. This is supported by the CD spectra of the recombinant minicollagen-1, which does not contain the positive ellipticity at 222 nm observed in the long collagens (Figure 2). Recent studies have suggested that this absorbance is a better indicator of proper collagen structure than the negative ellipticity at ~200 nm (Lopes et al., 2014). Comparing the TEM micrographs from the recombinant minicollagen-1 to those obtained previously for minicollagen-1 expressed and purified from a mammalian culture system is informative. Expression of minicollagen-1 in a mammalian cell-line yielded short, aligned rod like structures that had dimensions consistent with those expected from a trihelical collagen-like central structure (Engel et al., 2001). Additionally, amino acid analysis indicated that ~20% of the proline residues were hydroxylated. TEM micrographs of recombinant minicollagen-1 show bundles of oriented fibrils with no small rod-like structures evident (Figure 3). In fact, the structures formed by recombinant minicollagen-1 are more similar to those observed with cnidoin, a silk-like protein found with the minicollagens in the inner wall of the nematocyst (Balasubramanian et al., 2012; Beckmann et al., 2015). Like the minicollagens, cnidoin contains CRDs at the amino and carboxy termini of the protein and mammalian cell line expressed forms of cnidoin have shown a propensity to self-assemble into oriented fibrillar bundles (Beckmann et al., 2015). In addition, cnidoin has been shown to have elastomeric properties and adopts an extended disordered structure found in other elastomeric proteins (Beckmann et al., 2015). In the future, it will be interesting to see if the self-assembly into fibrillar bundles observed with cnidoin and recombinant minicollagen-1 are mediated by the terminal CRDs. Together, the data suggest that recombinant minicollgen-1 is not forming ordered secondary structures in solution, and does not form the trihelical quaternary structure observed with the long collagens or minicollagens expressed from metazoan sources. This is likely due, at least in part, to the inability of the *E. coli* expression system to proline hydroxylate recombinant minicollagen-1. Systems have been developed in bacteria to hydroxylate heterologously expressed collagen on proline (Ramshaw et al., 2019), so future studies will look at the role of proline hydroxylation in the self-assembly and stability recombinant minicollagen-1.

Regardless of whether recombinant minicollagen-1 adopts a trihelical structure, the protein demonstrates interesting properties that may make it useful in material applications, even in an unmodified form. The combination of CRDs, polyproline stretches and the short collagen-like central region make the minicollagen family a potentially unique material additive. Previous studies have shown that polyproline sequences can be distinguished from collagen-like sequences by environmental conditions, such as increased temperature and the existence of chaotropes (Tiffany and Krimm, 1972; Gutierrez-Cruz et al., 2001; Whittington et al., 2005; Lopes et al., 2014). The negative ellipticity demonstrated by the CD spectrum of minicollagen-1 is very heat stable (Figure 4), which is a property shared by polyproline peptides, but not collagen (Tiffany and Krimm, 1972; Gutierrez-Cruz et al., 2001). Alternatively, the negative ellipticity observed in recombinant minicollagen-1 CD spectra was very sensitive to the presence of urea, which is a property shared by collagen (Lopes et al., 2014). However, recombinant minicollagen-1 appears to be significantly more sensitive to urea than what has been reported for long collagen (Supplementary Figure 7). In contrast to what was observed with recombinant minicollagen-1, urea has been shown to increase the polyproline II helical structure of polyproline sequences (Gutierrez-Cruz et al., 2001; Whittington et al., 2005). Interestingly, the presence of DTT had a strong effect on the recombinant minicollagen-1, abrogating the negative ellipticity observed at 207 nm as was observed with urea treatment (Figure 5B). This is consistent with SDS-PAGE analysis where recombinant minicollagen-1 is observed to migrate faster when run under reducing conditions compared to the migration of the non-reduced protein (Supplementary Figure 8). The latter observations likely reflect the reduced ability of recombinant minicollagen-1 to bind SDS as compared to the more globular protein molecular weight standards, and indicate a change in protein conformation between oxidized and reduced states. The sensitivity of recombinant minicollagen-1 to reducing agents is distinct from what is observed with polyproline and collagen sequences, which demonstrate little to no change in secondary structure in the presence of reducing agents (Lopes et al., 2014). Therefore, recombinant minicollagen-1 appears to adopt a disordered conformation but has unique properties compared to other proline rich sequences, and can be distinguished by its sensitivity to reducing agents. This sensitivity may present a useful handle for the tuning of properties in recombinant minicollagen-1 based materials.

The above observations point to a dominant role of the terminal CRDs in the stabilization of recombinant minicollagen-1 secondary structure. The CRDs are present in numerous proteins involved in nematocyst biosynthesis and function (Ozbek et al., 2009; Balasubramanian et al., 2012). Recently, it was shown that the CRDs facilitate multimerization of proteins through direct interaction with each other, and that certain CRD sequences enable a higher interaction valency than others (Tursch et al., 2016). The dynamic nature of the CRD disulfide bonds has been demonstrated in the creation of re-codable surfaces where surface chemistry of a substrate can be written and re-written using the reversible nature of the CRD disulfide bonds (Gegenhuber et al., 2017). In the current study, the ability to utilize photocrosslinking in the creation of recombinant minicollagen-1 hydrogels through the mobilization of the CRD stored disulfides is a demonstration of how these domains may be used to create structured materials. Mechanical testing of the minicollagen-1 hydrogels yielded a compressive elastic modulus in the KPa range, comparable to the mechanical properties measured in other protein-based hydrogels created with irreversible covalent crosslinking (Supplementary Figure 9) (Partlow et al., 2014). This is important since the reversible nature of disulfide crosslinking allows for controlled degradation as well as the potential for dynamic reconfiguration. It is also significant that the recombinant minicollagen-1 demonstrates the ability to self-assemble into fibrils and fibril bundles with periodic spacing (Figure 3). Therefore, the recombinant minicollagen-1 presented in this study may be looked at as a naïve material precursor, with the potential for scalable expression and the ability to form hydrogels whose properties can be programmed with posttranslational modifications.

## Data Availability Statement

The datasets generated for this study are available on request to the corresponding author.

## Author Contributions

SF, PD, MC, AP, and SY performed processing, and characterization of materials. MC expressed and purified recombinant proteins. CH and JS performed TEM measurements. MG, PD, LD, MS, and RN conceived ideas and designed the experiments. All authors contributed to data analysis, discussions, and manuscript writing.

### Conflict of Interest

SF, JS, AP, and CH are employed by the company UES, Inc. The remaining authors declare that the research was conducted in the absence of any commercial or financial relationships that could be construed as a potential conflict of interest.

## Abbreviations

CRD: cysteine rich domain
IMAC: immobilized metal affinity chromatography
SDS-PAGE: sodium dodecyl sulfate polyacrylamide gel electrophoresis
DTT: dithiothreitol
EF-P: elongation factor P
CRB: chelating reducing buffer.
