# Supplementary material for: Disulfide Crosslinked Hydrogels Made From the Hydra Stinging Cell Protein, Minicollagen-1
Source: Front Chem. 2020 Jan 23;7:950. doi: 10.3389/fchem.2019.00950 (PMC6989532; doi:10.3389/fchem.2019.00950)
Supplement: Supplementary file 1 [file Data_Sheet_1.pdf]

Non-tagged minicollagen-1

MDANPCGSYCPSVCAPACAPVCCYPPPPPPPPPPPPPPPPPPPPPPPAPLP
GNPGPPGRPGPPGAPGPAGPPGLPGPPGPPGAPGQGGLPGQPAPPPPP
CPPVCVAQCVPTCPQYCCPAKRK

**GPP**	**36%**
**GXP**	**86%**
**GPY**	**7%**

His-tagged minicollagen-1

MGSSHHHHHHSSGLVPRGSHMDANPCGSYCPSVCAPACAPVCCYPPPPP
PPPPPPPPPPPPPPPPPPAPLPGNPGPPGRPGPPGAPGPAGPPGLPGPPG
PPGAPGQGGLPGQPAPPPPPCPPVCVAQCVPTCPQYCCPAKRK

Supplemental Figure 1. The primary structures of non-tagged and hexahistidine-tagged minicollagen-1 variants expressed in bacteria. The relative amount of the different G-X-Y repeats in the collagen-like domain is indicated in red lettering on the side.

Supplemental Figure 2

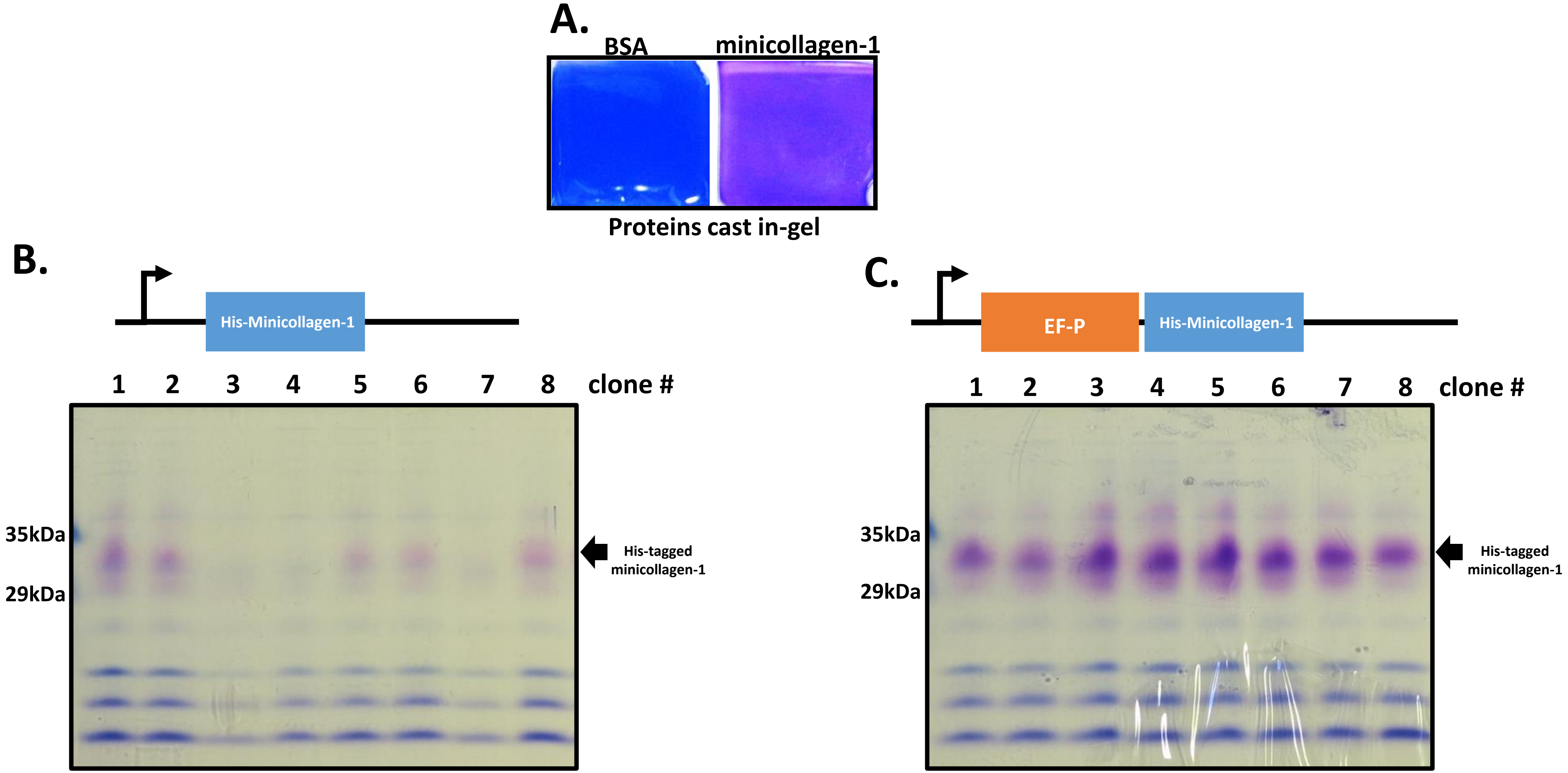

Supplemental Figure 2. (A) Coomassie R-250 staining of polyacrylamide gels cast with the indicated proteins (BSA-bovine serum albumin). (B and C) Coomassie R-250 staining of IMAC pulldowns from extracts of bacterial cultures from eight separate clones of bacteria transformed with hexa-histidine-recombinant minicollagen-1 alone (B) and co-expressed with EF-P (C). The organization of the individual open reading frames are indicated above relative to the promoter (bent arrow).

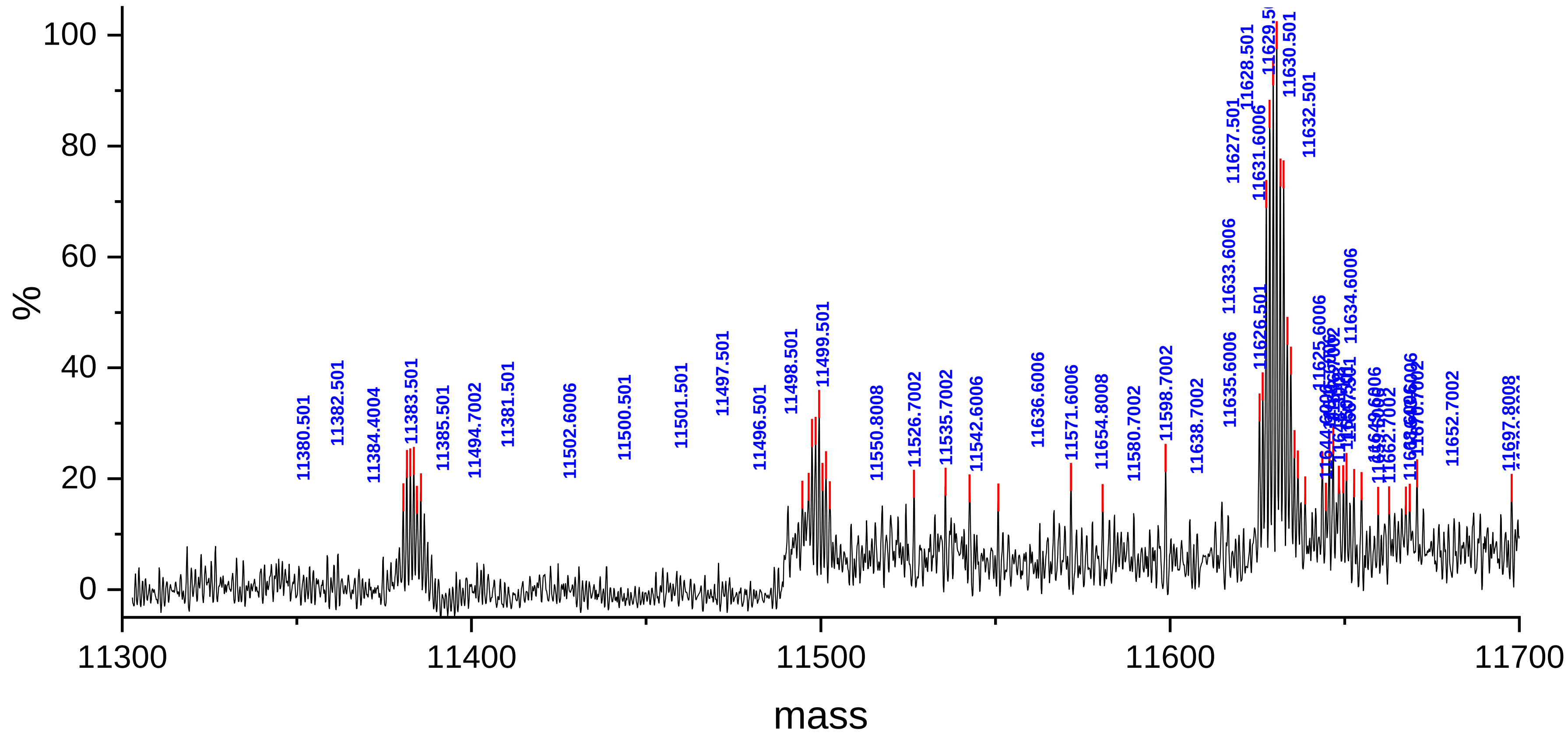

Supplemental Figure 3. Mass Spectrometry of non-tagged recombinant minicollagen-1 purified from bacteria, showing deconvolved masses across expected theoretical mass range which is calculated as 11641.70 da.

Supplemental Figure 4

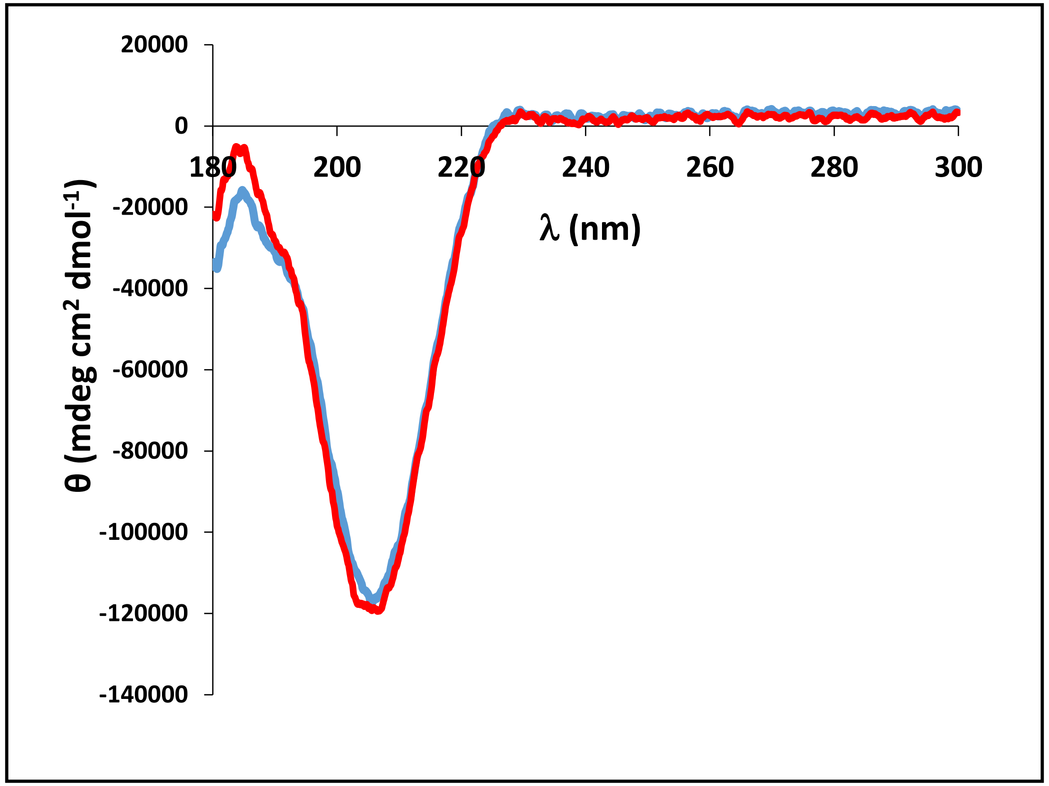

Supplemental Figure 4. Circular dichroism (CD) spectroscopy performed on solutions (1.4 µM) of purified, bacterially expressed hexa-histidine-tagged (blue line) and non-tagged (red line) recombinant minicollagen-1. The spectra were taken after the minicollagen-1 samples were serially diluted in deionized water from 86 µM to 28 µM to 5.8 µM to the final concentration of 1.4 µM. This procedure differs from the other CD spectra where the 1.4 µM concentration was directly diluted from the 86 µM stock.

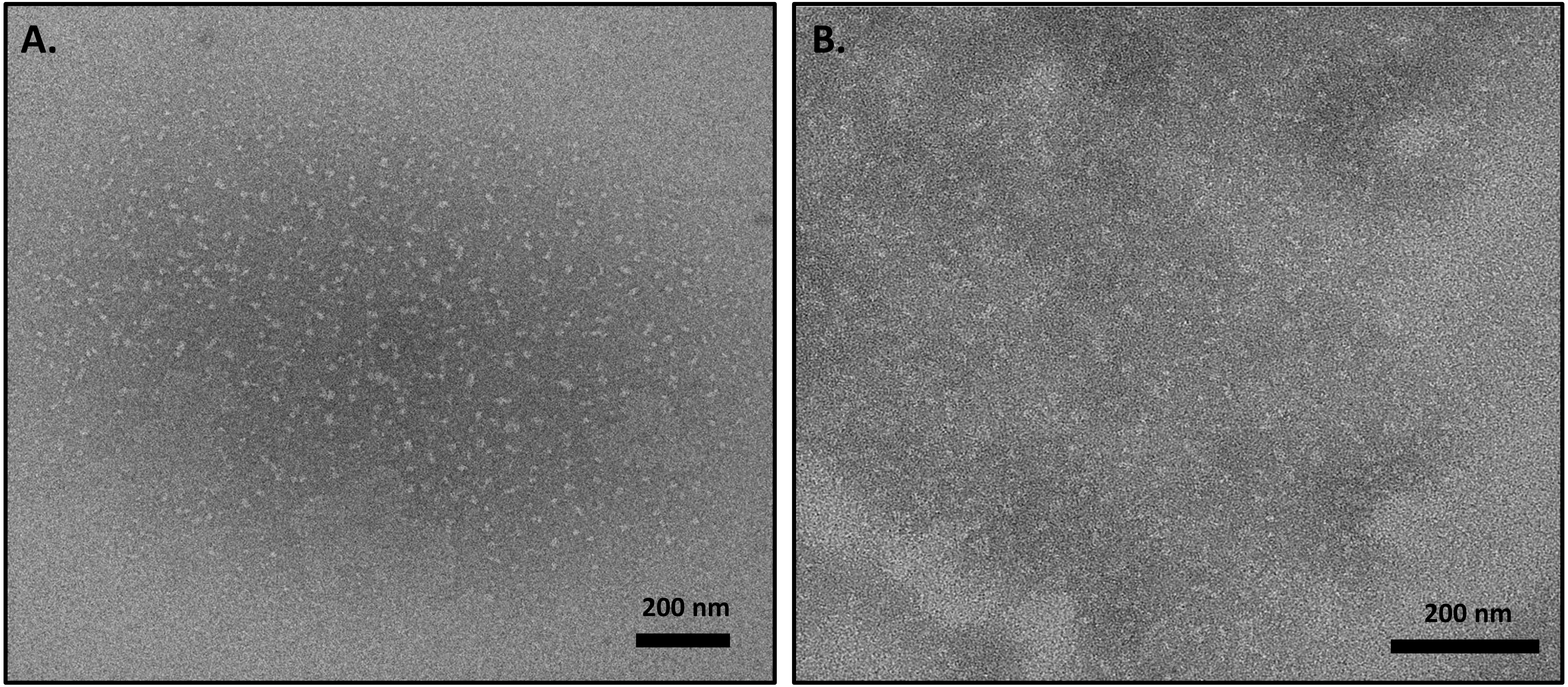

Supplemental Figure 5. (A and B) TEM analysis of dilute (0.1 mg/ml), non-tagged recombinant minicollagen-1 purified from bacteria. The scale bar distance is indicated on the figure.

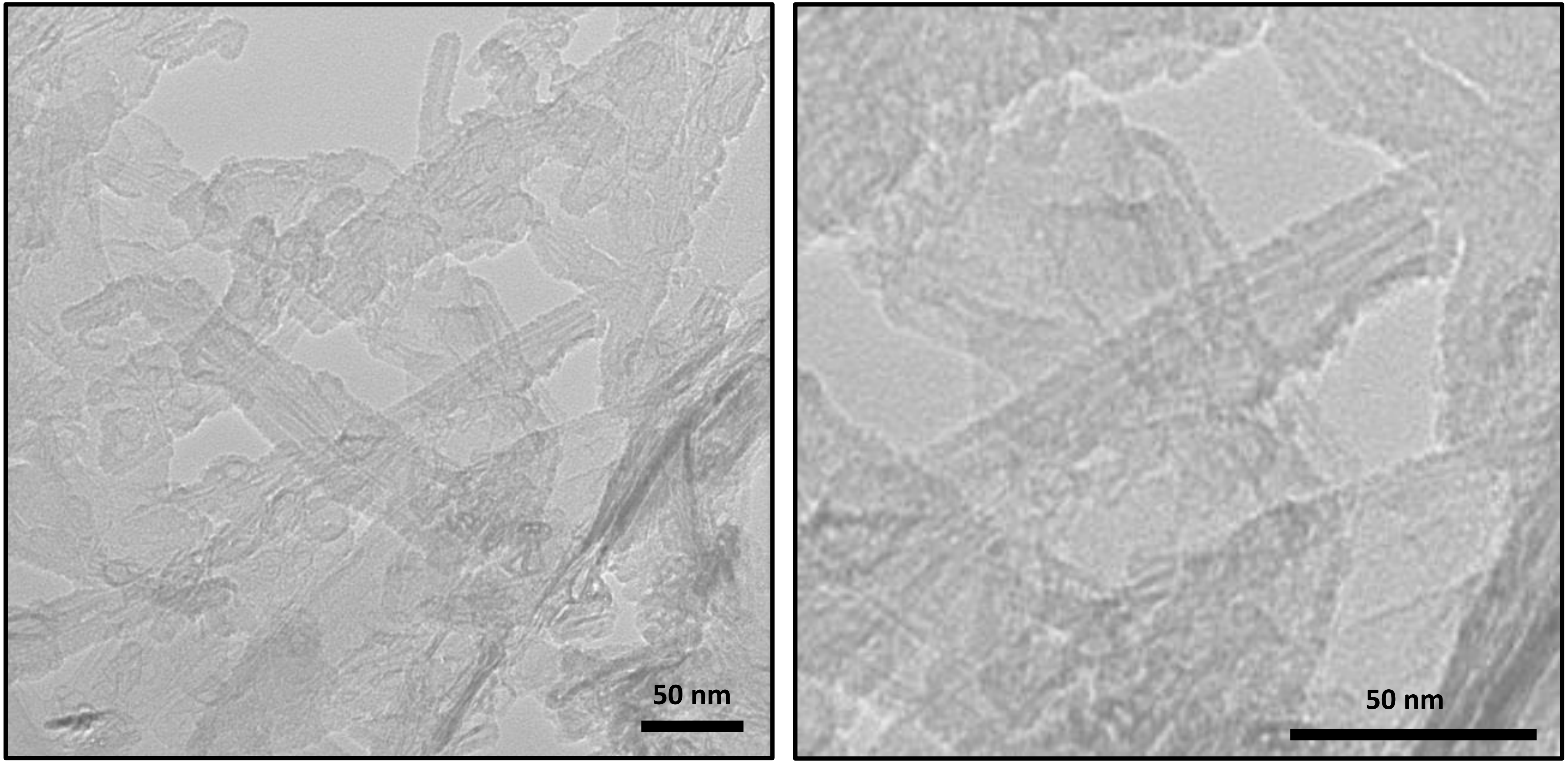

Supplemental Figure 6. TEM analysis of concentrated (1 mg/ml), hexa-histidine-tagged recombinant minicollagen-1 purified from bacteria. The scale bar distance is indicated on the figure.

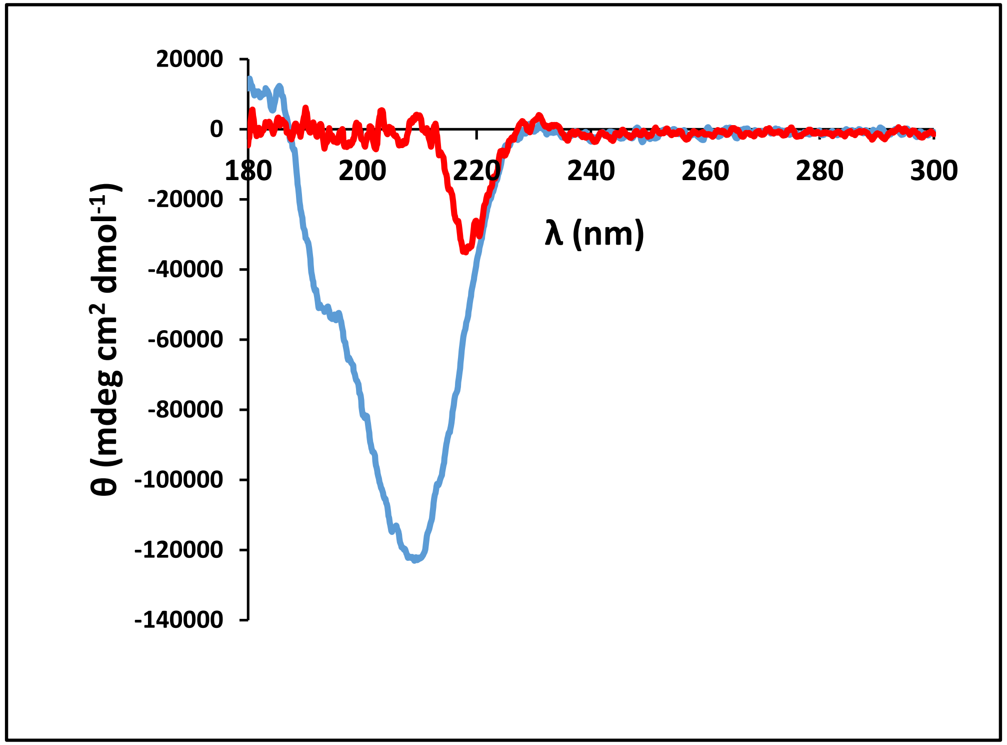

Supplemental Figure 7. CD spectroscopy of non-tagged recombinant minicollagen-1 (1.4 μM) in the absence (blue line) and presence (red line) of 1M urea.

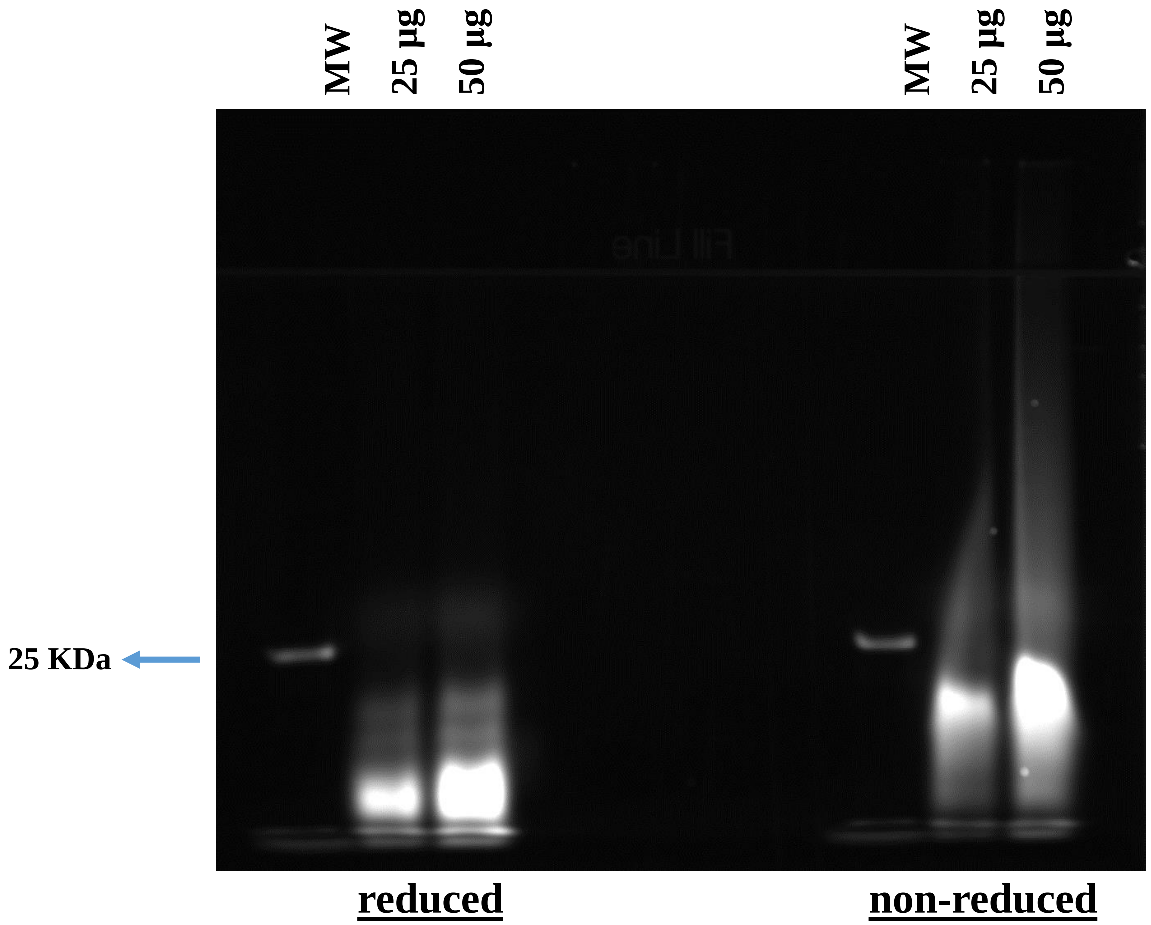

Supplemental Figure 8. SDS-PAGE of rhodamine labelled non-tagged minicollagen-1 under reducing and non-reducing conditions.

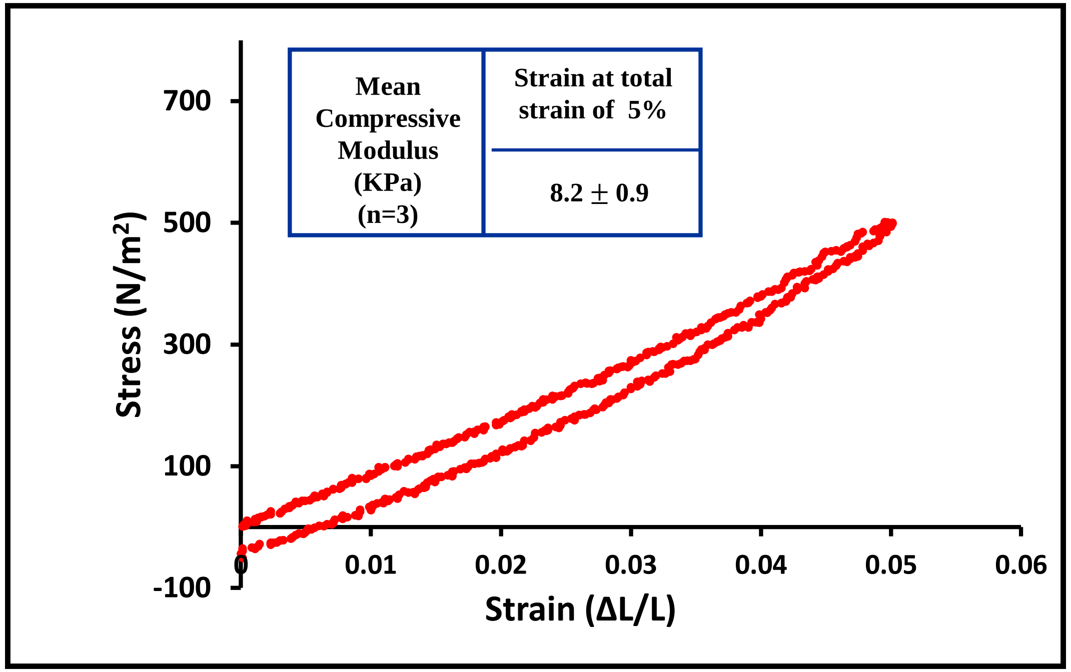

Supplemental Figure 9. Stress versus strain curve for compression and then recovery of a Nvjp-1 hydrogel (chemically crosslinked with horseradish peroxidase).
